# Super-Enhancers in the Regulation of Gene Transcription: General Aspects and Antitumor Targets

**DOI:** 10.32607/actanaturae.11067

**Published:** 2021

**Authors:** A. V. Bruter, M. D. Rodionova, E. A. Varlamova, A. A. Shtil

**Affiliations:** Institute of Gene Biology, Russian Academy of Sciences, Moscow, 119334 Russia; Blokhin National Medical Research Center of Oncology, Moscow, 115478 Russia; ITMO University, Saint-Petersburg, 197101 Russia

**Keywords:** transcription, super-enhancers, transcriptional protein kinases, targeted therapy, tumors

## Abstract

Super-enhancers (genome elements that activate gene transcription) are DNA regions with an elevated concentration of transcriptional complexes. These multiprotein structures contain, among other components, the cyclin-dependent kinases 8 and 19. These and other transcriptional protein kinases are regarded as novel targets for pharmacological inhibition by antitumor drug candidates.

## INTRODUCTION

The template synthesis of molecules (and gene transcription in particular) is one of the most essential processes in nature. This evolutionary conservative mechanism is found in all organisms, without exception: from viruses to higher mammals. Its biological role consists of transmitting and consolidating genetic information from the template macromolecule in the offspring. The cornerstone role of transcription is not limited to the “normal” processes, such as ontogenesis and phylogenesis, speciation, biodiversity, control of heredity, etc. Deepening our understanding of the molecular mechanisms of transcription allows us to grasp its fundamental significance in pathological processes. Today, it is impossible to interpret the etiology and pathogenesis of diseases without an analysis of the regulation of gene expression in a pathological site. It appears reasonable to assert that differential gene expression (changes in the set of functioning genes, activity (intensity) and temporal regulation of expression compared to the physiological pattern) defines the essence of a disease as a “transcriptional imbalance.”

Our modern approach to therapy (targeted manipulation with specific transcription mechanisms) is currently rooted in this understanding. These mechanisms in mammalian cells are unusually diverse, interchangeable, and they remain insufficiently understood; in addition, a number of them remain unsusceptible to pharmacological regulation (the so-called non-druggable targets). Therefore, targeted “transcriptional therapy” is only in its first steps.

Which structural and functional elements of the transcription apparatus can be influenced to regulate gene expression? Which mechanisms should one regulate and how is this problem solved in terms of the spatial organization of transcription? Research focused on the regulatory regions of genes (promoters and enhancers) is necessary as, among the functions of these regions, are ensuring proper localization of multiprotein transcriptional complexes, transcription initiation, and regulation of the transcription rate. Since protein kinases are perhaps the most common targets in modern drug design, it is no coincidence that, among the various mechanisms of gene expression regulation, transcriptional kinases (a separate class of serine/threonine phosphotransferases) are emerging as the study object, the potential therapeutic target.

This review analyzes genomic elements where the presence of the transcriptional machinery is especially potent: super-enhancers and the proteins associated with them (transcription factors, cofactors, and protein kinases). We consider these elements as the structural and functional units of the transcription apparatus, and as therapeutic targets in tumor cells.

## SUPER-ENHANCERS: SPECIAL ENHANCERS?

**Definition of the concept**

The concept of super-enhancers was first formulated in a study focused on the regulation of gene expression in embryonic stem cells. Whyte *et al*. [1] disclosed a number of the traits of the regulatory regions of the genes whose active expression is associated with the maintenance of the undifferentiated pluripotent state (*Oct4*, *Sox2*, *Nanog*, *Klf4*, *Esrrb*, miR-290-295, etc.) These genomic regions differ from the conventional enhancers in terms of length and distance from the regulated gene, as well as in terms of the number and set of transcription factors associated with them. Oct4, Sox2, Nanog, Klf4, and Esrrb proved to be the prevailing transcription factors (the occupancy of the latter two factors in conventional enhancers is particularly different from that in super-enhancers). They are the key transcription factors that support, and can even induce, the pluripotent state of embryonic cells, as well as Med1, a component of the Mediator complex. The identified areas were named super-enhancers. An important feature of super-enhancers was discovered already in that first study: when the level of transcription factors in the cell changes (e.g., when the amount of Oct4 or the Mediator complex partially decreases), transcription of the corresponding genes stops, while transcription of the genes regulated by conventional enhancers changes insignificantly [1].

An attempt to provide a generalized definition of a “super-enhancer” makes it necessary to draw a distinction between these regions of the genome and conventional enhancers. This boundary turns out to be conditional (see below). The definition of super-enhancers is empirical and is based on two criteria. Super-enhancers include genomic regions with the following features: (1) regions containing extended (up to 12.5 kb) groups of enhancers; (2) regions with abnormally high binding of a certain set of transcription factors (these typically are transcription factors that are essential for the physiology of cells of a given type: Oct4, Sox2, and Nanog in embryonic stem cells; MyoD, a crucial tissue-specific transcription factor in muscle cells [2], etc.) and cofactors. In practice, these two structural criteria correlate with two functional criteria: a high expression level of the genes regulated by super-enhancers and an abrupt change in the expression level in response to small changes in the concentration of transcription factors [1, 3].

Although several thousand enhancers regulate the expression of thousands of genes, only a few hundred super-enhancers regulate the expression of the genes whose products are particularly important for cells of a given type [1, 4-6]. In addition, some super-enhancers function according to the positive feedback mechanism: super-enhancers regulate the expression of the genes encoding transcription factors that enhance the transcription of the genes regulated by super-enhancers. These genes include *Oct4*, *Sox2*, *Nanog*, *Klf4*,* Esrbb*, and *Prdm14 *[6].

It is noteworthy that during evolution, super-enhancers were acquired by many of the genes playing a key role in cell biology, but not by the so-called housekeeping genes, which are characterized only by a consistently high expression level. Super-enhancers act as the end target of the main signal cascades more often than conventional enhancers do. In addition to an increased level of binding to the transcription factors (Oct4, Sox2, Nanog, etc.) that regulate the maintenance of the undifferentiated state of embryonic stem cells, increased binding of super-enhancers to the transcription factors closing the main signaling pathways (TCF3 (the WNT signaling pathway), SMAD3 (the TGF-β signaling pathway), STAT1 (the JAK-STAT signaling pathway), and STAT3 (LIF)) was also detected [7, 8].

**Identification of super-enhancers in the genome**

**Fig. 1:**
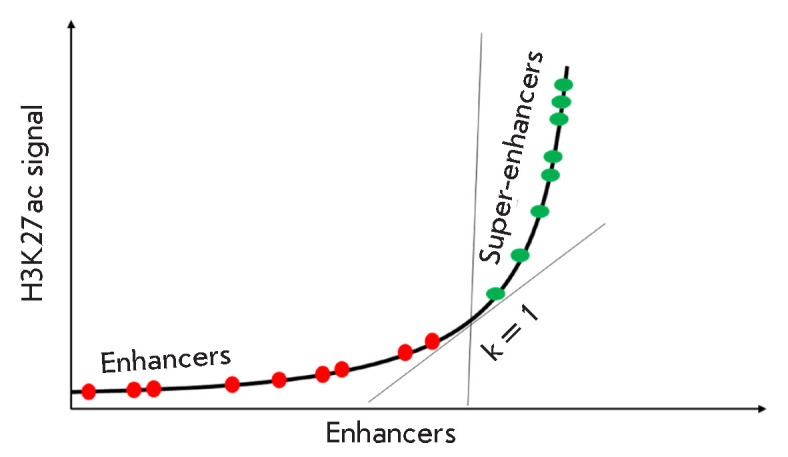
Conventional enhancers and super-enhancers. The distribution of enhancers depending on the number of bound H3K27Ac molecules is shown. Adapted from [3, 10]

The most common method used to identify active super-enhancers is based on the characteristic epigenetic state inherent to active enhancers: monomethylation, instead of trimethylation, of lysine at position 4 of histone 3 (H3K4me1; it allows one to distinguish between enhancers’ active promoters) and acetylation of lysine at position 27 of histone 3 (it “marks” active enhancers as opposed to inactive regulatory elements) [1, 3, 9]. At the first (experimental) stage, H3K27ac chromatin regions are immunoprecipitated, with subsequent sequencing of the DNA fragments associated with them. The obtained data are processed using bioinformatics (see [1, 4]). The DNA sequences found during sequencing are compared with the corresponding genome, and the regions that appear repeatedly (the so-called peaks) are identified. Peaks separated from each other by less than 12.5 kb are combined into single extended enhancers. The density of H3K27ac in the enriched sites is then normalized to the average density of H3K27ac for a given genomic region, and the enhancers are arranged in increasing order of enrichment. The resulting curve is characterized by an abrupt increase within the region of high enrichment in histone 3 with acetylated lysine-27. The enhancers contained in this area are referred to as super-enhancers; the criterion is that the enhancer is located on the plot (*Fig. 1*) to the right from the point at which the derivative of the enrichment function equals 1 [10].

Along with enrichment in histone “marks,” other molecular criteria for active transcription can be used to identify super-enhancers: sensitivity to DNase I, increased binding of transcription factors (Oct4, Sox2, Nanog, etc.), the presence of the activators Med1 and p300 [1, 3, 6]. The SEdb database [11] contains more than 300,000 super-enhancers, from 542 samples obtained from human cell lines. The differences in the number of super-enhancers between individual lines of non-tumor and tumor cells are specified. This database makes it possible to analyze, in detail, the nucleotide sequences of super-enhancers and identify binding sites for the transcription factors, polymorphisms, etc.

Super-enhancers identified by any of these methods consist of only a few single enhancers, and about 15% of the super-enhancers consist of just one enhancer [12]. Such an unclear empirical definition, which is also based on a conditional choice of distinction between conventional enhancers and super-enhancers, allows one to raise the following question: are super-enhancers actually a separate class of regulatory elements or are they a particularly effective type of enhancers?

## FUNCTIONING OF SUPER-ENHANCERS

The difference between conventional enhancers and super-enhancers is clearly manifested in the nature of the dependence of the transcription activity ensured by the regulatory element and the number of transcription factors and cofactors associated with it. This dependence is linear for conventional enhancers, while, for super-enhancers, it acquires an “all or nothing” form [1, 13] resembling the dependences describing phase transitions in the framework of statistical thermodynamics. In practice, this manifests itself as a high sensitivity of super-enhancers to changes in conditions. Deletion of a small area or a reduced concentration of one of the cofactors (BRD4, CDK7) can completely inactivate the super-enhancer [4, 6, 14, 15]. A detailed analysis of these mechanisms is provided below.

**Fig. 2:**
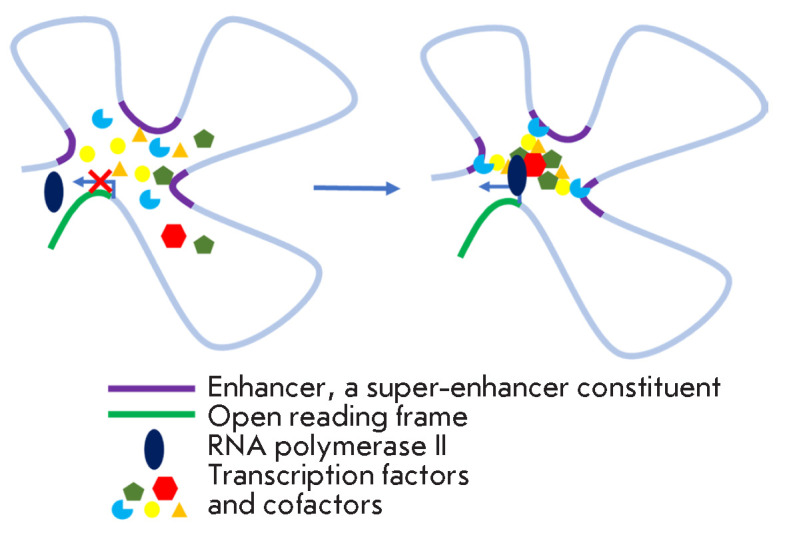
The phase separation model of the structure and function of super-enhancers. Transcription factors and coactivators interact with different regions of the super- enhancer and with each other. The high intensity of these interactions leads to phase separation of DNA-protein transcription complexes and an abrupt transcription activation. *Left*: the situation before the start of interaction and separation. mRNA synthesis by RNA polymerase II does not occur. *Right*: after separation and transcriptional activation. Adapted from [14]

**Fig. 3:**
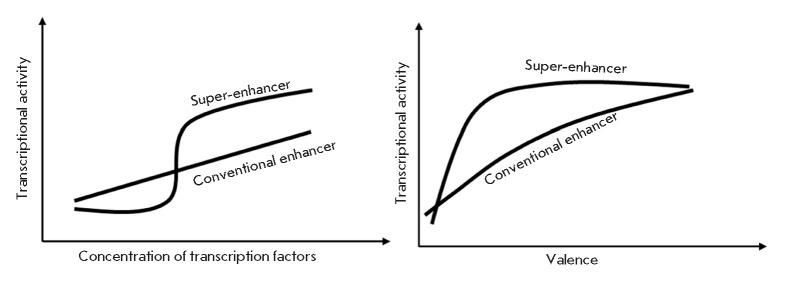
*Left*: General dependence of transcriptional activity on the concentration of transcription factors for enhancers and super-enhancers. *Right*: Dependence of enhancer and super-enhancer activity on valence (i.e., the number of available intermolecular interactions according to the phase separation model). The regular enhancer is modeled by a system consisting of 10 molecules, whereas the super-enhancer is modeled by 50 molecules. Adapted from [14]

A phase separation model was proposed to explain the regularities of super-enhancer function [13]. The high concentration of intensely interacting molecules gives rise to a membraneless organelle that is phase-separated from the rest of the nucleus. The model takes into account two numerical indicators: the number of molecules in a given volume (DNA, histones, transcription factors, and cofactors) (it is assumed that on average it is equal to 10 for a conventional enhancer and 50 for a super-enhancer) and the “valence” of these molecules (a number describing how many interactions are available to the molecule). In this model, transcriptional activity depends on the percentage of molecules interacting with each other at a given time (*Fig. 2*, *Fig. 3*). The state of phase separation occurs when almost all molecules interact (i.e., the fraction of interacting molecules approaches unity). In this state, the transcriptional activity is at its maximum. The valence of the molecules in the system can grow (e.g., during chromatin remodeling and activation in the enhancer or super-enhancer region). Mathematical modeling has shown that the transcriptional activity of a conventional enhancer depends linearly on the valence of the system, and for a super-enhancer, at relatively low valence values, phase separation occurs and transcriptional activity increases abruptly almost to a maximum.

According to this model, valence decreases upon inhibition of a cofactor or deletion of the binding site. In the case of a super-enhancer, it causes a significant drop in transcriptional activity from the maximum to the minimal value.

In addition to DNA and protein molecules, the complex also contains enhancer RNAs (eRNAs): non-coding RNAs transcribed from the enhancers. Among them, there are short-lived short RNAs without poly(A) regions that can be transcribed in both directions, and longer ones with poly(A) regions (transcribed only in the 5‘ → 3’ direction). eRNAs are involved in the organization of promoter–nhancer interactions: they increase the strength of the binding of transcription factors to DNA, recruit and activate cofactors, and shorten the transcriptional pause. Super-enhancers express eRNA at a higher level than conventional enhancers do; in addition, eRNAs are more often expressed from the super-enhancers rather than from conventional enhancers [10]. eRNAs can be involved in the activation of the expression of the corresponding gene but can also activate other genes, including those located on other chromosomes, thus spreading the impact of the enhancer (distant regulation of the genome) [16].

## SUPER-ENHANCERS IN THE REGULATION OF NORMAL AND PATHOLOGICAL PROCESSES

Super-enhancers are much more likely to act as regulators of the key processes in normal cells and pathological processes compared to conventional enhancers [6]. The IgH 3′RR super-enhancer located in the 3′-regulatory region of the *IgH *locus on chromosome 14 of the human genome regulates recombination in B cells (in particular, V(D)J recombination in B1 cells [17] and isotype switching, depending on the external signal in B2 cells [18]). Another element that is important in this process is the super-enhancer of the *Aicda *locus*. *Enzymes belonging to the TET family and ensuring demethylation of this super-enhancer are required for isotype switching [19]. Conversion of adipocytes from brown fat to white fat is accompanied by the activation of a super-enhancer associated with the gene encoding the nuclear receptor PPARγ [20]. Furthermore, because of the activation of the super- enhancer, renin synthesis is induced by renal cells that do not synthesize renin under normal conditions. A super-enhancer is activated only in the offspring of embryonic renin-producing cells [21]. CircRNA (circNfix), regulated by a super-enhancer that is specific to mature cardiomyocytes [22], precludes the division of mature cardiomyocytes, and its suppression improves tissue regeneration after an experimental myocardial infarction in mice.

Point mutations in the noncoding regions of the genome account for about 90% of all disease-related mutations (according to genome-wide association studies (GWAS)). Such mutations are more common in super-enhancers than they are in conventional enhancers. This conclusion was bolstered by comparing the super-enhancers in different types of cells from the same patient: in the abnormal focus and outside of it. Mutations (single-nucleotide polymorphisms, SNPs) in super-enhancers are associated with Alzheimer’s, systemic lupus erythematosus, type 1 diabetes mellitus, etc. Thus, several SNPs have been found in the super-enhancer of the *BIN1 *gene, whose increased expression is associated with the risk of developing Alzheimer’s. In the case of type 1 diabetes mellitus, an increased amount of mutations was found in super-enhancers in T-helper cells. Polymorphisms associated with systemic lupus erythematosus were found to be concentrated in the super-enhancers of the key genes for B-cells [6].

Super-enhancers can also be epigenetically activated in response to external stimuli. Thus, during inflammation, activation of the transcription factor NF-κB in endothelial cells can lead to the formation of active super-enhancers and maintenance of a high expression of genes whose products promote the adhesion of leukocytes (*SELE*, *VCAM1*), as well as chemokine CCL2. Super-enhancers have been activated due to binding of the acetylated form of NF-κB to BRD4. No super-enhancers were activated upon BRD4 inhibition in [23].

**Super enhancers in tumor cells**

The high level of expression of oncogenes in malignant cells may be due to the emergence of a new super-enhancer. Accelerated proliferation of malignant cells is regulated by signaling cascades. The proliferation intensity of colon adenocarcinoma cells (HCT116 line) depends on the activation of the Wnt signaling pathway; in this lineage, a super-enhancer is activated at the *c-MYC *locus*. *Along with this, increased binding of the transcription factor TCF4, an effector of the Wnt cascade, to the super-enhancer was discovered. A similar regulation mechanism was encountered in the cells of estrogen-dependent breast cancer [7].

The emergence of super-enhancers in tumor cells occurs according to the same mechanisms as any changes in gene expression. Both genetic (chromosomal translocations [6], amplification [24, 25, 26, 27], deletions, insertions [28], and point mutations) and epigenetic (activation of oncogen expression [4, 6, 11, 29, 30, 31 ] or reduced expression of anti-oncogenes [8, 31, 32, 33]) mechanisms can participate in malignant cell transformation. In the first variant, the emergence of new super-enhancers and the disappearance of previously existing ones is possible, as well as the transfer of potential oncogenes under the control of active super-enhancers that are unusual for them. Epigenetic regulation is represented by activation or deactivation of the corresponding super- enhancers.

**Fig. 4:**
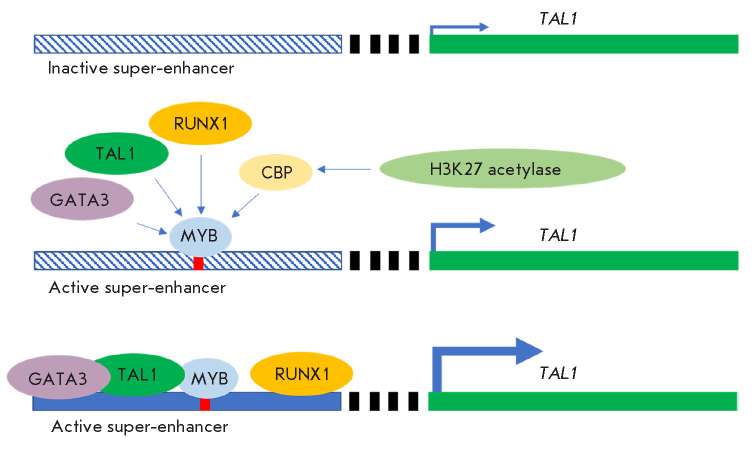
Insertion of the Myb transcription factor binding site activates the super-enhancer-driven expression of the* TAL1 *oncogene. The DNA-bound Myb recruits the cyclic AMP response element binding protein (CBP) and its partner, histone acetylase H3K2, to activate chromatin in the super-enhancer region. Additional transcription factors are also recruited

The *c-MYC *locus, especially, frequently acquires super-enhancers during carcinogenesis. In multiple myeloma cells, the super-enhancer regulating the gene expression of the *igH *locus appears in the *c-Myc *locus via translocation [6]. In some patients with acute T-cell leukemia, reduplications were found in the non-coding region, consistent with the super-enhancer regulating the *c-Myc *gene. The amplified fragment ensures the Notch-dependent functioning of the super-enhancer [25]. The amplified region was associated with the proteins of the SWI/SNF complex, which remodels chromatin and is key in the proliferation of tumor cells [34]. Focal amplifications (copying of a small region of the genome, followed by the transferring of copies to arbitrary genomic regions) of super-enhancers in the 3’-regulatory region of the *c*-*Myc *gene were found in lung and endometrial tumors [26]. In a similar manner, focal amplification of a super-enhancer in the 3’-regulatory region of the *KLF4 *gene and its increased expression were found in squamous cell carcinoma of the head and neck [27]. In T-cell leukemias, a small (2–2 bp) insertion forming a binding site for the Myb transcription factor mediates the formation of an active 8-kb super-enhancer, thus recruiting additional transcription factors [28] (*Fig. 4*).

An important genetic mechanism of malignant cell transformation is represented by a violation of the boundaries of topologically associating domains (TADs), which are chromosomal segments approximately 1 Mbp long that are transcriptionally isolated from each other. The fragments of one TAD interact with each other much more often than the fragments of different TADs. Division into these domains is an evolutionarily conserved process that probably arose to prevent the long-range interaction of enhancers and super-enhancers with “foreign” promoters. The boundaries between the TADs are the binding sites of the CTCF transcription repressor (CCGCGNGGNGGCAG) and the CTCF–ohesin protein complexes associated with them. Mutations in the genes encoding cohesin and CTCF, as well as in their DNA-binding sites, are often present in transformed cells [9]. Small deletions were found at the TAD boundaries in the Jurkat cell line (CD4**+**8**+ **thymocytes). When reproducing these deletions in epithelial cells (HEK293 line) using the CRISPR/Cas9 system, a super-enhancer-dependent activation of the oncogenes *TAL1 *and *LMO1 *was detected [35].

Changes in the nucleotide sequences of super-enhancers may both affect their binding to proteins and also cause changes in the sequences and the number of eRNA molecules. Certain eRNAs associated with “oncogenic” super-enhancers have oncogenic properties themselves. These eRNAs are involved in the regulation of the key processes: proliferation, apoptosis, autophagy, epithelial–esenchymal transition, and angiogenesis [16].

The bromodomain protein BRD4, which binds to acetylated lysine residues in histones (i.e., to active chromatin), plays an important role in the epigenetic regulation of super-enhancer activity. A study focused on diffuse large B-cell lymphomas showed that onethird of the BRD4 molecules in a cell are concentrated in super-enhancers. Expression of the corresponding genes was found to be very sensitive to the pharmacological inhibition of BRD4 [14]. A low-molecular-weight inhibitor of BRD4, compound JQ1, reduced the expression of super-enhancer-dependent genes, in particular, the *c*-*Myc *oncogene*, *in myeloma cells [4]. The high level of expression of *c-Myc *in colon cancer cells (HCT116 and DLD1 lines) is also supported by a super-enhancer. Knockdown of the *BRD4*, *MED12, *and *MED13/13L* genes decreased enhancer*-*dependent gene expression and inhibited the proliferation of colorectal cancer cells [29].

An unexpected variant of epigenetic activation of super-enhancers was discovered in the study of B-cell infection with the Epstein–Barr virus. In infected cells, this virus synthesizes its own transcription factors, EBNA2, 3A, 3C, and EBNA-LP, and activates some cellular ones (RelA, RelB). These transcription factors form the active super-enhancers that regulate the* c*-*Myc, MIR155, IKZF3, *and *Bcl-2 *genes that are crucial to cell survival. The activation of super-enhancers is sensitive to BRD4 inhibition: it was blocked by the compound JQ1 [36].

Super-enhancers also regulate the differentiation status of tumor stem cells; this fact can be used in elaborating therapeutic strategies. For maintaining the pluripotent state of glioma stem cells, the ELOVL2 (elongation of the very long chain fatty acids protein 2) protein, whose expression in these cells is particularly high and is triggered by the epigenetic activation of the corresponding super-enhancer, is important. ELOVL2 plays a key role in the synthesis of polyunsaturated fatty acids (components of the plasma membrane) and is also involved in the signaling from the epidermal growth factor receptor (EGFR). Selective inactivation of the ELOVL2 super-enhancer by dCas9-KRAB leads to a post-transcriptional decrease in the EGFR level [30]. Loss of functional activity by the B-cell transcriptional regulator Ikaros is associated with a poor prognosis in patients with B-cell acute lymphoblastic leukemia. This is because Ikaros is required for the terminal differentiation of rapidly proliferating B-cell progenitors. The “two-facedness” of Ikaros is rather interesting: it maintains the inactive state of chromatin in the regions of super-enhancers of the genes whose expression determines the undifferentiated state of B-cells, but it also maintains active chromatin in the super-enhancers of the genes whose products are important for differentiation [31].

## SUPER-ENHANCERS AS A FOCUS OF THERAPEUTIC TARGETING

This example illustrates a situation where the malignant potential of a cell is ensured by the inactivation of the expression of anti-oncogenes dependent on super- enhancers rather than by the high expression of oncogenes caused by super-enhancers. In this regard, therapeutic strategies aimed at reactivating anti-oncogene super-enhancers appear reasonable. The possibility of implementing this strategy is analyzed below.

As mentioned above, super-enhancers make it possible to fundamentally alter the transcriptional program, even in response to a relatively weak stimulus. Malignant transformation is often associated with the emergence (formation) or activation of an existing, but non-functioning, super-enhancer. Therefore, super-enhancers and the associated proteins are gaining interest as targets for the development of anticancer drugs. It is hoped that a therapeutic effect will be achieved at relatively low concentrations of such drugs (see below).

Two classes of proteins associated with super-enhancers are considered as therapeutic targets: proteins with a bromodomain (primarily BRD4) and the cyclin- dependent protein kinases CDK4/6, CDK7, CDK8, and CDK12/13.

**Proteins containing a bromodomain**

Proteins carrying a conserved lysine-binding amino acid sequence, the bromodomain, ensure the functioning of super-enhancers by maintaining the active state of chromatin through interaction with the acetylated lysine residues in chromatin proteins. As a result of this interaction, transcription factors and RNA polymerase II are recruited to super-enhancers. Inhibition of proteins carrying the bromodomain can lead to chromatin inactivation.

BRD4 inhibitors (small-molecule compounds JQ1 and I-BET151) have shown encouraging results in preclinical models of acute myeloid leukemia and multiple myeloma. Tumor growth retardation, as well as suppression of *Myc *expression and downstream transcription programs, was observed [9]. A number of compounds that inhibit BRD2/3/4/T by competitive binding are currently undergoing clinical trials [37]. The ABBV-075 inhibitor (Mivebresib) was tested on 10 patients with acute myeloid leukemia resistant to standard therapy and/or recurrent forms of the disease; one of the patients achieved complete remission, the number of blast cells in bone marrow in four patients was reduced at least twofold, and good treatment tolerance was observed. Combination therapy is also promising [38].

**Cyclin-dependent protein kinases**

The cyclin-dependent kinases CDK4 and CDK6 play a key role in the phase change of the G1-S cell cycle. The CDK4/6 inhibitors palbociclib, ribociclib, and abemaciclib have been included in hormone-sensitive HER2-negative breast cancer protocols as monotherapy. As part of combination therapy, CDK4/6 inhibitors are undergoing clinical trials for other types of breast cancer [39]. Selective inhibitors of CDK4/6 have a cytostatic effect and cause death of Ewing sarcoma cells in culture and *in vivo*, and they also reduce the expression of a number of genes dependent on super-enhancers (in particular, cyclin D1) [40].

A special group of cyclin-dependent protein kinases does not participate in the regulation of cell cycle phases but functions as a structural and functional component of the transcription apparatus. In particular, such “transcriptional” protein kinases include CDK7, CDK8 and its paralog CDK19 (CDK8/19), as well as CDK9 and CDK12/13 [41]. CDK7 is a component of the TFIIH transcription-initiating complex; it mediates the phosphorylation of the C-terminal domain of RNA polymerase II and transcription initiation. CDK9 within the p-TEFb complex also regulates the transition to elongation by phosphorylation of the C-terminal domain of RNA polymerase II [41]. CDK12 and CDK13 directly activate mRNA elongation and processing [42].

THZ1, an inhibitor of CDK7 (and, to some extent, CDK9 and CDK12) [43, 44], reduces transcription in cells of various tissue origins; the transformed cells were found to be sensitive to low THZ1 concentrations. The compound suppressed the oncogenes associated with super-enhancers, in particular, at the *c*-*Myc *locus [45]. Clinical trials of a more selective inhibitor of CDK7, the SY5609 compound, were launched in 2020 [46].

Super-enhancer-dependent expression of the* RUNX1*, *MYB*, *TAL1, *and *GATA3 *oncogenes decreases in Jurkat cells under the influence of THZ531, an inhibitor of CDK12 [47]. Since the first specific inhibitors of CDK12 have been synthesized recently, their clinical trials are yet to be started. However, it has been shown in cell cultures and tumor models in mice that inhibition of CDK12 has a pronounced effect on osteosarcoma, liver, breast, and ovarian tumors, as well as neuroblastoma [48].

CDK8 plays a special role in transcription regulation. This serine/threonine protein kinase, in cooperation with cyclin C (CCNC), the MED12, and MED13 proteins, forms the regulatory CDK module of a crucial transcriptional complex: Mediator. The components of this complex are conserved in all eukaryotes. It is important to understand that CDK8/19, unlike other CDKs, does not regulate phase transitions in the cell cycle [49]. The main function (but not the only one) of CDK8/19 is to regulate the phosphorylation of the C-terminal domain of RNA polymerase II at the serine- 2 and serine -5 residues of the heptapeptide repeat constituting this domain. This phosphorylation was shown in a cell-free system. In cells, this event is necessary at different stages of transcription (initiation, pause release, and elongation of the primary transcript); however, the role of CDK8/19 in this phosphorylation needs to be proved experimentally. In contrast to CDK7 and CDK9, which function on all promoters, CDK8/19 is involved only in the regulation of the activity of RNA polymerase II on actively transcribed genes (inducible genes and genes functioning in the development of the organism) [50, 51, 52, 53]. The selectivity of expression activation indicates that CDK8/19 is one of the key mechanisms of transcriptional reprogramming. This unique feature has been the subject of extensive research in recent years.

Transcriptional reprogramming is not vital for an adult organism under homeostatic conditions; longterm inhibition of CDK8/19 has no phenotypic manifestations. Genetic (mediated by the Cre/Lox system) knockout of the *cdk8 *gene also has no significant manifestations in adult mice [54]. However, reprogramming of transcription is necessary for the development of the organism: knockout of *cdk8 *in mice is lethal at the preimplantation embryo stage [55], and null mutations in the genes encoding the cdk8 or ccnc proteins in* Drosophila melanogaster *leads to death at the late third instar larva and prepupal stages [56, 57].

Importantly, the *CDK8 *gene knockout and pharmacological inhibition of kinase activity have different effects on the general patterns of gene expression, which indicates two fundamentally different mechanisms of CDK8/19 action: those dependent on and independent of kinase function.

Is there a connection between CDK8/19 and super- enhancers? In immunoprecipitation experiments, an increased presence of CDK8 in the regions of individual super-enhancers was detected. According to the RNA sequencing data, two-thirds of the genes whose expression is affected by CDK8 inhibition are the genes regulated by super-enhancers. Among super-enhancers whose relationship with CDK8 was established in both of the aforementioned experimental systems, there were super-enhancers of the genes encoding the transcription factors Nanog, Oct3/4, and SOX2, as well as a significant number of super-enhancers of the genes regulated by the Wnt signaling pathways [32].

## SUPER-ENHANCERS AND CDK8/19: THE TARGETS OF ANTITUMOR ACTION

Transcriptional reprogramming is fundamental in the development of many pathological processes, especially tumor ones. Deregulation of CDK8/19 is often encountered in tumors in which CDK8 is involved in the activation of important signaling pathways mediated by Wnt/β-catenin [58], NF-κB [51], TGF-β [59], HIF1α [51], or the estrogen receptor [41] regulating the response to changes in the serum concentration [50]. CDK8 was found to be an oncoprotein associated with the development of colorectal cancer [47], tumors of the pancreas [60] and mammary glands [52, 61, 62, 63], and melanoma [64]. CDK8 is responsible for the phenotype of cancer stem cells [65].

Since CDK8/19 inhibition is practically safe in an adult organism, it is promising to use CDK8/19 as therapeutic targets [41, 66, 67]. Compounds belonging to various chemical classes acting as a platform for the proposed pharmacological blockers of CDK8/19 kinase activity and the so-called degraders for complete elimination of these proteins (overcoming of the kinase-independent function) are being intensively studied [68, 69]. The issues related to inhibitor selectivity have been discussed in reviews and original studies [53, 70, 71, 72, 73].

Cortistatin A is a relatively selective inhibitor of CDK8/19 kinase activity. Inhibition of these protein kinases by cortistatin A in acute myeloid leukemia cells (MOLM-14 line) increased the expression of the “antitumor” genes controlled by super-enhancers (*CEBPA*, *IRF8*, *IRF1, *and *ETV6*), which slowed down cell proliferation. Meanwhile, the expression of 20% of the genes associated with super-enhancers increased, while only a 3% increase in expression was observed for the genes with the conventional enhancers. CDK8 was found to be associated with the super-enhancers of all activated genes (versus 67% of activated genes with the conventional enhancers). There were only three inhibited genes regulated by the super-enhancers (1% of all the genes regulated by super-enhancers). These ratios allow [33] one to infer that the proteins associated with super-enhancers are the direct targets of cortistatin A in MOLM-14 cells.

Treating these cells with the I-BET151 compound, an inhibitor of BRD4, reduced the expression of the genes regulated by super-enhancers, although the result (an antitumor effect) was the same as that observed after exposure to cortistatin A. Proliferation of tumor cells is likely to depend on the “dosage” of gene expression determined by super-enhancers. It is noteworthy that the effects were not summarized for I-BET151 and cortistatin A used together, and that the changes in the gene expression profile were in full alignment with those caused by I-BET151 alone. BRD4 is probably required for transcription activation in response to cortistatin A [33].

Acute myeloid leukemia cells are sometimes characterized by constitutive activation of the JAK-STAT signaling pathway. The STAT1 transcription factor is one of the main targets of CDK8 kinase activity. The content of the phosphorylated (transcriptionally competent) form pSTAT1S727 is increased in the super-enhancer regions [8]. It turns out that CDK8-dependent phosphorylation of STAT1 is required for rapid proliferation of leukemia cells. Exposure to cortistatin A led to a slowdown in the proliferation and activation of the super-enhancer-dependent expression of the GATA1, GATA2, and ID2 transcription factors mediating proliferation slowdown or cessation, as well as the activation of the megakaryocyte-specific PLEK, CFLAR, and UBASH3B factors. As a result, transition of cells from the stem state to the differentiated state and inhibition of proliferation were observed [8].

CDK8/19 inhibitors based on modified pyridines slowed the proliferation of colorectal cancer cells [32]. The gene expression patterns changed in the same fashion as when a number of super-enhancers were activated. Under the influence of CDK8/19 inhibitors, tumor cells went from the stem phenotype to a differentiated state. As mentioned above, this process is associated with the activation of super-enhancers. Activation of the *c-Myc *oncogene, which is also regulated by a super-enhancer, is consistent with the concept of increased super-enhancer-dependent expression upon CDK8 inhibition. However, despite the activation of* c*-*Myc*, the inhibitors were found to exhibit an overall moderate antitumor effect [32].

Kuuluvainen *et al*. [29] attempted to devise a way to selectively inactivate the super-enhancers ensuring high oncogene expression in colorectal cancer cells. The reduction in the CDK8 level by RNA interference led to an integral decrease in the expression of the super-enhancer-regulated genes, but no selective decline in the expression of the super-enhancer-regulated oncogenes was observed. This decrease was caused by knockdown of the *MED12 *and *MED13/13L* genes [29].

In the examples discussed above, CDK8/19 inhibition did not affect the activated enhancers of oncogenes but led to the activation of the super-enhancers of the anti-oncogenes [8, 32, 33]. The antitumor effect is attributed to the restoration of a differentiated phenotype and slowdown in cell proliferation. Hence, the use of CDK8/19 inhibitors in the treatment of certain tumor types should be considered a super-enhancer- mediated restoration of normal gene expression in malignant cells. Meanwhile, the pathological process is not limited to transcription disorders: post-transcriptional and post-translational events also play a role.

Early stages of clinical trials of CDK8/19 inhibitors are currently underway. For instance, SEL120 compounds are being tested as candidate drugs against acute myeloid leukemia, and BCD-115 are studied for possible treatment of HER2-negative estrogen-dependent breast cancer. Trial identifiers, as well as the analysis of the causes of the toxicity of CDK8/19 inhibitors, were presented in [71].

## CONCLUSION. SUPER-ENHANCERS AS THERAPEUTICALLY SIGNIFICANT ELEMENTS OF THE GENOME

Detailed studies of the structural and functional features of genome organization have made it possible to formulate the concept of super-enhancers as regions with an increased content of transcription complexes. It is not surprising that these regions are important in pathogenesis: the molecular mechanisms of diseases are associated, in one way or another, with dysregulation of gene transcription. Super-enhancers acquire a special role in tumor biology: uncontrolled proliferation of transformed cells and their evasion of therapeutic action (chemotherapy and radiation therapy) are caused by both transcription activation and by adaptive changes in their gene expression profile. Consequently, super-enhancers (the DNA regions carrying multiprotein transcriptional “machines”) become targets of antitumor action.

The question related to the prospects of the low-molecular- weight chemical modulators of transcriptional CDKs in tumor therapy is especially important. The effectiveness of the first CDK inhibitors turned out to be insufficient, and general resorptive toxicity was high. Subsequently, more selective inhibitors of individual transcriptional protein kinases were obtained: THZ1 for CDK7, THZ531 for CDK12/13, and palbociclib and ribociclib for CDK4/6. These compounds have a pronounced antitumor effect in clinical situations and are becoming parts of treatment regimens [74, 75].

CDK8/19s are of interest as a unique target: the special selectivity of transcription reprogramming offers a chance to replace the currently used toxic drugs with well-tolerated agents inhibiting this mechanism. Although occurring in all age groups, acute myeloid leukemia is especially common in patients over 60 years of age. The currently used treatment regimens are difficult to tolerate due to the cardio- and myelotoxicity; the likelihood of early relapse is high (within the first year) [76, 77]. In our experiments, the selective CDK8/19 inhibitor senexin B caused the death of acute myeloid leukemia cells (line MV-4-11) when used at significantly lower concentrations than cytosar, one of the main chemotherapy drugs used for treating this disease. Senexin B produced the indicated effect at concentrations that were non-toxic to non-tumor cells. In the culture of chronic myeloid leukemia cells, senexin B increased the antitumor effect of targeted inhibitors of chimeric tyrosine kinase Bcr-ABL [78], thus broadening the possibilities of CDK8/19 inhibition in the therapy of blood cancers. The outcomes with chemotherapy for colorectal cancer (especially metastatic disease [79, 80]) remain unsatisfactory; therefore, the results of studies focused on this tumor and demonstrating the effectiveness of an inactivation of the Mediator complex components seem rather promising [29, 32, 81].

While it may be difficult to interpret super-enhancers as special “independent” regulatory elements of the genome from a general biology point of view, their practical importance as “accumulators” of transcription complexes for studying a pathogenesis and developing personalized therapy seems undeniable. This strategy involves identifying the role of a specific transcriptional mechanism in the patient (the transcriptional “portrait”) and targeting the established mechanism.

## References

[1] Whyte W., Orlando D., Hnisz D., Abraham B., Lin C., Kagey M., Rahl P., Lee T., Young R. (2013). Cell..

[2] Peng X., So K., He L., Zhao Y., Zhou J., Li Y., Yao M., Xu B., Zhang S., Yao H. (2017). Nucleic Acids Research.

[3] Tang F., Yang Z., Tan Y., Li Y. (2020). NPJ Precis. Oncol..

[4] Lovén J., Hoke H., Lin C., Lau A., Orlando D., Vakoc C., Bradner J., Lee T., Young R. (2013). Cell..

[5] Parker S., Stitzel M., Taylor D., Orozco J., Erdos M., Akiyama J., Bueren K., Chines P., Narisu N., Black B. (2013). Proc. Natl. Acad. Sci. USA..

[6] Hnisz D., Abraham B., Lee T., Lau A., Saint-André V., Sigova A., Hoke A., Young R. (2013). Cell..

[7] Hnisz D., Schuijers J., Lin C.Y., Weintraub A., Abraham B., Lee T., Bradner J., Young R. (2015). Molecular Cell.

[8] Nitulescu I., Meyer S., Wen Q., Crispino D., Lemieux M., Levinee R., Pelish H., Shair M. (2017). EBioMedicine..

[9] Thandapani P. (2019). Pharmacol. Ther..

[10] Peng Y., Zhang Y. (2018). Animal Model Exp. Med..

[11] (2020). The comprehensive human Super-Enhancer database. Date of the application: 05.08.2020..

[12] Pott S., Lieb J. (2014). Nat. Genet..

[13] Hnisz D., Shrinivas K., Young R., Chakraborty A., Sharp P. (2017). Cell..

[14] Chapuy B., McKeown M., Lin C., Monti S., Roemer M., Qi J., Rahl P., Sun H., Yeda K., Doench J. (2013). Cancer Cell..

[15] Ball B., Abdel-Wahab O. (2018). Trends Pharmacol. Sci..

[16] Tan Y., Li Y., Tang F. (2020). Mol. Cancer..

[17] Ghazzaui N., Issaoui H., Saintamand A., Oblet C., Carrion C., Denizot Y. (2018). Blood Adv..

[18] Santos J., Braikia F., Oudinet C., Joana M., Dauba A., Khamlichi A. (2019). Proc. Natl. Acad. Sci. USA..

[19] Lio C., Shukla V., Samaniego-Castruita D., González-Avalos E., Chakraborty A., Yue X., Schatz D., Rao A. (2019). Sci. Immunol..

[20] Loft A., Forss I., Siersbæk M., Schmidt S., Larsen A., Madsen J., Pisani D., Nielsen R., Aagaard M., Mathison A. (2014). Genes Dev..

[21] Martinez M., Medrano S., Brown E., Tufan T., Shang S., Bertoncello N., Guessoum O., Adli M., Belyea B., Sequeira-Lopez M., Gomez R. (2018). J. Clin. Invest..

[22] Huang S., Li X., Zheng H., Si X., Li B., Wei G., Li C., Chen Y., Chen Y., Liao W. (2019). Circulation..

[23] Brown J., Lin C., Duan Q., Griffin G., Federation A., Paranal R., Bair S., Newton G., Lichtman A., Kung A. (2014). Molecular Cell.

[24] Iwakawa R., Takenaka M., Kohno T., Shimada Y., Totoki Y., Shibata T., Tsuta T., Nishikawa R., Noguchi M., Sato-Otsubo A. (2013). Genes Chromosomes Cancer..

[25] Herranz D., Ambesi-Impiombato A., Palomero T., Schnell S., Belver L., Wendorff A., Xu L., Castillo-Martin M., Llobet-Navás D., Cordon-Cardo C. (2014). Nat. Med..

[26] Zhang X., Choi P., Francis J., Imielinski M., Watanabe H., Cherniack A., Meyerson M. (2015). Nat. Genet..

[27] Zhang X., Choi P., Francis J., Gao G., Campbell J., Ramachandran A., Mitsuishi Y., Ha G., Shih J., Vazquez F. (2018). Cancer Discov..

[28] Mansour M., Abraham B., Anders L., Berezovskaya A., Gutierrez A., Durbin A., Etchin J., Lawton L., Sallan S., Silverman L. (2014). Science..

[29] Kuuluvainen E., Domènech-Moreno E., Niemelä E., Mäkelä T. (2018). Mol. Cell Biol..

[30] Gimple R., Kidwell R., Kim L., Sun T., Gromovsky A., Wu Q., Wolf M., Lv D., Bhargava S., Jiang L. (2019). Cancer Discov..

[31] Hu Y., Zhang Z., Kashiwagi M., Yoshida T., Joshi I., Jena N., Somasundaram R., Emmanuel A., Sigvardsson M., Fitamant J. (2016). Genes Dev..

[32] Clarke P., Ortiz-Ruiz M., TePoele R., Adeniji-Popoola O., Box G., Court W., Czasch S., Bawab S., Esdar C., Ewan K. (2016). eLife..

[33] Pelish H., Liau B., Nitulescu I., Tangpeerachaikul A., Poss Z., Da Silva D., Caruso B., Arefolov A., Fadeyi O., Christie A. (2015). Nature.

[34] Shi J., Whyte W., Zepeda-Mendoza C., Milazzo J., Shen C., Roe J., Minder J., Mercan F., Wang E., Eckersley-Maslin M. (2013). Genes Dev..

[35] Hnisz D., Weintraub A., Day D., Valton A., Bak R., Li C., Goldmann J., Lajoie B., Fan Z., Sigova A. (2016). Science..

[36] Zhou H., Schmidt S., Jiang S., Willox B., Bernhardt K., Liang J., Johannsen E., Kharchenko P., Gewurz B., Kieff E. (2015). Cell Host Microbe..

[37] Alqahtani A., Choucair K., Ashraf M., Hammouda D., Alloghbi A., Khan T., Senzer N., Nemunaitis J. (2019). Future Sci OA..

[38] Borthakur G., Wolff J., Aldoss I., Hu B., Dinh M., Torres A., Chen X., Rizzieri D., Sood A., Odenike O. (2018). J. Clin. Oncol..

[39] Sobhani N., D’Angelo A., Pittacolo M., Roviello Z., Miccoli A., Corona S., Bernocchi O., Generali D., Otto T. (2019). Cells..

[40] Kennedy A., Vallurupalli M., Chen L., Crompton B., Cowley G., Vazquez F., Weir A., Tsherniak A., Parasuraman S., Kim S. (2015). Oncotarget..

[41] Chou J., Quigley D., Robinson T., Feng F., Ashworth A. (2020). Cancer Discov..

[42] Greenleaf A. (2019). Transcription..

[43] Kwiatkowski N., Zhang T., Rahl P., Abraham B., Reddy J., Ficarro S., Dastur A., Amzallag A., Ramaswamy S., Tesar B. (2014). Nature.

[44] Rix U., Superti-Furga G. (2009). Nat. Chem. Biol..

[45] Chipumuro E., Marco E., Christensen C., Kwiatkowski N., Zhang T., Hatheway C., Abraham B., Sharma B., Yeung C., Altabef A. (2014). Cell..

[46] A Study of SY 5609, a Selective CDK7 Inhibitor, in Advanced Solid Tumors. Date of the application: 05.08.2020..

[47] Zhang T., Kwiatkowski N., Olson C., Dixon-Clarke S., Abraham B., Greifenberg A., Ficarro S., Elkins J., Liang Y., Hannett N. (2016). Nat. Chem. Biol..

[48] Liang S., Hu L., Wu Z., Chen Z., Liu S., Xu X., Qian A. (2020). Cells..

[49] Galbraith M., Donner A., Espinosa J. (2010). Transcription..

[50] Donner A., Ebmeier C., Taatjes D., Espinosa J. (2010). Nat. Struct. Mol. Biol..

[51] Galbraith M., Allen M., Bensard C., Wang X., Schwinn M., Qin B., Long H., Daniels D., Hahn W., Dowell R. (2013). Cell..

[52] McDermott M., Chumanevich A., Lim C., Liang J., Chen M., Altilia S., Oliver D., Rae J., Shtutman M., Kiaris H. (2017). Oncotarget..

[53] Chen M., Liang J., Ji H. (2017). Proc. Natl. Acad. Sci. USA..

[54] McClelandM. I.O., SoukupT. I.O., Liu S., Esensten J., de Sousa E. Melo., Yaylaoglu M., Warming S., Roose-Girma M., Firestein R. (2015). J. Pathol..

[55] Westerling T., Kuuluvainen E., Makela T. (2007). Mol. Cell Biol..

[56] Loncle N., Boube M., Joulia L., Boschiero C., Werner M., Cribbs D., Bourbon H. (2007). EMBO J..

[57] Xie X., Hsu F., GaoX. I.O., Xu W., Ni J., Xing Y., Huang L., Hsiao H., Zheng H., Wang C. (2015). PLoS. Biol..

[58] Firestein R., Bass A., Kim S., Dunn I., Silver S., Guney I., Freed E., Ligon A., Vena N., Ogino S. (2008). Nature.

[59] Alarcon C., Zaromytidou A., Xi Q., Gao S., Yu J., Fujisawa S., Barlas A., Miller A., Manova-Todorova K., Macias M. (2009). Cell..

[60] Xu W., Wang Z., Zhang W., Qian K., Li H., Kong D., Li Y., Tang Y. (2015). Cancer Lett..

[61] Porter D., Farmaki E., Altilia S., Schools G., West D., Chen M., Chang B., Puzyrev A., Lim C., Rokow-Kittell R. (2012). Proc. Natl. Acad. Sci. USA..

[62] Broude E., Gyorffy B., Chumanevich A., Chen M., Mc-Dermott M., Shtutman M., Catroppo J., Roninson I. (2015). Curr. Cancer Drug Targets..

[63] Xu D., Li C., Zhang X., Gong Z., Chan C., Lee S., Jin G., Rezaeian A., Han F., Wang J. (2015). Nat. Commun..

[64] Kapoor A., Goldberg M., Cumberland L., Ratnakumar K., Segura M., Emanuel P., Menendez S., Vardabasso C., Leroy G., Vidal C. (2010). Nature.

[65] Adler A., McCleland M., Truong T., Lau S., Modrusan Z., Soukup T., Roose-Girma M., Blackwood E., Firestein R. (2012). Cancer Research.

[66] Menzl I., Witalisz-Siepracka A., Sexl V. (2019). Pharmaceuticals (Basel)..

[67] Xi M., Chen T., Wu C., Gao X., Wu Y., Luo X., Du K., Yu L., Cai T., Shen R., Sun H. (2019). Eur. J. Med. Chem..

[68] Solum E., Hansen T., Aesoy R., Herfindal L. (2020). Bioorg. Med. Chem..

[69] Hatcher J., Wang E., Johannessen L., Kwiatkowski N., Sim T., Gray N. (2018). ACS Med. Chem. Lett..

[70] Philip S., Kumarasiri M., Teo T., Yu M., Wang S. (2018). J. Med. Chem..

[71] Chen M., Li J., Liang J., Thompson Z., Kathrein K., Broude E., Roninson I. (2019). Cells..

[72] Chen W., Ren X., Chang C. (2019). ChemMedChem..

[73] He L., Zhu Y., Fan Q., Miao D., Zhang S., Liu X., Zhang C. (2019). Bioorg. Med. Chem. Lett..

[74] Jia Q., Chen S., Tan Y., Li Y., Tang F. (2020). Exp. Mol. Med..

[75] Sengupta S., George R. (2017). Trends Cancer..

[76] Brinda B., Khan I., Parkin B., Konig H. (2018). J. Cell. Mol. Med..

[77] Tan Y., Wu Q., Zhou F. (2020). Crit. Rev. Oncol. Hematol..

[78] Khamidullina A., Tatarskiy V., Yastrebova M., Nuzhina J., Ivanova E., Lim C.U., Chen M., Broude E., Roninson I., Shtil A. (2020). Proc. EACR conference «A Matter of Life and Death: Mechanisms, Models and Therapeutic Opportunities ». Italy,2020.

[79] Feng S., Yan P., Zhang Q., Li Z., Li C., Geng Y., Wang L., Zhao X., Yang Z., Cai H., Wang X. (2020). Int. J. Colorectal Dis..

[80] Kotani D., Kuboki Y., Horasawa S., Kaneko A., Nakamura Y., Kawazoe A., Bando H., Taniguchi H., Shitara K., Kojima T. (2019). BMC Cancer..

[81] Liang J., Chen M., Hughes D., Chumanevich A., Altilia S., Kaza V., Lim C., Kiaris H., Mythreye K., Pena M. (2018). Cancer Research.

